# Clinical and genetic characteristics of children with sodium taurocholate cotransporting poly-peptide deficiency

**DOI:** 10.3389/fped.2026.1784165

**Published:** 2026-06-10

**Authors:** Lina Du, Mengyao Zhou, Jing Yang, Min Du, Maolin Jiang, Lijing Xiong

**Affiliations:** Department of Pediatric Gastroenterology, Chengdu Women and Children’s Central Hospital, School of Medicine, University of Electronic Science and Technology, Chengdu, Sichuan, China

**Keywords:** case series, hyperbileacidemia, hypercholanemia, NTCP, *SLC10A1* gene

## Abstract

**Introduction:**

Sodium taurocholate cotransporting polypeptide deficiency (NTCPD) is an autosomal recessive disorder caused by *SLC10A1* gene mutations. This study analyzed the clinical features, genetic spectrum, and natural history of NTCPD in a single-center pediatric cohort to guide clinical management.

**Methods:**

We conducted a retrospective review of clinical and genetic data from children diagnosed with NTCPD between September 2020 and August 2024, and performed a 12-month observational follow-up.

**Results:**

This study included 19 children who exhibited heterogeneous clinical presentations. The participants were categorized into a jaundice group (*n* = 9) and an Incidental Hypercholanemia (IH) group (*n* = 10). Compared to the IH group, patients in the jaundice group were significantly younger (*p* = 0.003), predominantly exhibited indirect hyperbilirubinemia, and had markedly elevated *γ*-glutamyl transferase (*γ*-GT) levels (*p* = 0.001). All patients achieved normalization of liver function parameters during follow-up. Genetic analysis revealed biallelic *SLC10A1* variants in all patients, including the pathogenic p.S267F variants and other variants currently classified as variants of uncertain significance. The homozygous c.800C > T (p.Ser267Phe) variant was the most prevalent (14/19). Four novel variants were identified: c.101T > C, c.551delT, c.896T > C and c.654_674dup.

**Discussion:**

This study expands the clinical data of Chinese children with NTCPD, confirming the predominance of the c.800C > T mutation and revealing phenotypic heterogeneity alongside generally favorable outcomes. Elevated *γ*-GT levels suggest potential biliary system involvement in the pathophysiology of the disease. Future multi-center collaborations and extended follow-up are warranted to further elucidate genotype-phenotype correlations and long-term prognostic outcomes.

## 1 Introduction

Bile acids (BAs) play a critical role in the digestion and absorption of lipids, cholesterol, and fat-soluble nutrients such as vitamin E. They also act as signaling molecules regulating diverse physiological functions, including anti-inflammatory, anti-cancer, anti-viral, and immunomodulatory effects (1). The metabolism of BAs depends on the enterohepatic circulation: liver-synthesized BAs are secreted into the bile, stored and concentrated in the gallbladder, and released into the intestine upon food intake. Approximately 95% of BAs are actively reabsorbed in the terminal ileum and returned to the liver via the portal vein, after which they are re-secreted into the biliary system (2, 3). However, when BA concentrations reach abnormally high levels, they become cytotoxic. Elevated BA levels can disrupt cell membranes, increase reactive oxygen species production, and trigger apoptosis and necrosis (4, 5).

The sodium-taurocholate co-transporting polypeptide (NTCP), encoded by the *SLC10A1* gene located on chromosome 14 in humans, is a solute carrier protein composed of 349 amino acids. It is specifically expressed on the basolateral membrane of hepatocytes and serves as a key transporter (6). Its primary physiological role is to mediate the efficient uptake of conjugated bile salts from portal blood into the liver, representing the initial and rate-limiting step in the enterohepatic circulation of BAs (6). Impaired NTCP function leads to reduced hepatic uptake of BAs; and elevated serum BA levels, and potentially resulting in hepatocyte injury and systemic symptoms.

NTCP deficiency (NTCPD) is an autosomal recessive disorder caused by loss-of-function mutations in the *SLC10A1* gene, leading to defective NTCP activity. The first case of NTCPD was reported by Vaz et al. (7) in 2014, with subsequent case reports emerging globally. However, affected children exhibit significant clinical heterogeneity, and the correlation with conventional liver biomarkers is complex (8, 9). Therefore, this study analyzes the clinical and genetic characteristics of 19 children diagnosed with NTCPD at our center, aiming to improve clinicians’ understanding of the disease and provide insights for its comprehensive management.

## 2 Methods

### 2.1 Ethics statement

This study was approved by the Ethics Committee of Chengdu Women's and Children's Central Hospital [Approval No. (2023)12], and written informed consent was obtained from the guardians of all participants.

### 2.2 Patient cohort

A retrospective analysis was conducted on clinical data and genetic test results of children with NTCPD who were hospitalized in the Department of Gastroenterology at our institution from September 2020 to August 2024. A total of 19 children were enrolled in this study. General and clinical data, including sex, age, weight, family history, birth history, and past medical history, were collected from these patients. Additionally, clinical manifestations, liver function results, and genetic mutation findings were also analyzed.

Based on clinical manifestations at consultation, the children were divided into the jaundice group (*n* = 9) and the incidental hypercholanemia group (IH, *n* = 10). There were no overlapping clinical symptoms between the two groups. The IH group consisted of children diagnosed with pneumonia or acute bronchitis, whereas the jaundice group comprised children presenting with delayed resolution of neonatal jaundice. No liver biopsy was performed in this cohort. For patients with hyperbilirubinemia, hemolysis-related markers -including reticulocyte count, lactate dehydrogenase, and hemoglobin levels – were assessed. All patients underwent a 12-month follow-up after discharge, during which clinical symptoms and liver function were monitored. No specific treatments were administered to these patients during the follow-up period. Laboratory parameters were primarily analyzed at our hospital's Department of Laboratory Medicine. Some clinical and biochemical data were obtained from medical records provided by the parents during referral to our center. The biochemical data from other hospitals were standardized when necessary.

The diagnostic criteria for NTCPD were as follows: (1) persistently elevated serum BA levels; and (2) confirmation of biallelic pathogenic mutations in the *SLC10A1* gene in either a homozygous or compound heterozygous state. Liver dysfunction was defined as alanine aminotransferase (ALT) levels exceeding two times the upper limit of normal. Inclusion criteria for NTCPD were: (1) age < 18 years; (2) elevated BAs, with or without abnormalities in other liver function parameters; (3) completion of genetic metabolic testing. Exclusion criteria included: (1) incomplete medical records; (2) loss to follow-up; (3) presence of cholestatic diseases due to biliary atresia, congenital choledochal cyst, confirmed infection(including CMV, EBV, HBV, HIV, and syphilis), drug-induced liver injury, or immune-related factors.

### 2.3 Molecular analysis

3 mL of peripheral venous blood was collected from each pediatric patient and their parents into EDTA-anticoagulated tubes. After thorough mixing, samples were promptly delivered to Maijinuo Co., Ltd (Beijing China) for targeted genetic testing using the Metabolic Liver Disease Gene Panel V6 (Project No. M030V2). The panel is a targeted next-generation sequencing (NGS) panel encompassing 611 genes associated with inherited metabolic liver diseases, cholestatic disorders, and related conditions. The panel covers coding exons and flanking intronic regions, with a mean sequencing depth of 300×. The *SLC10A1* gene (associated with NTCP deficiency) is included in this panel (Supplementary Table 1). Following the identification of candidate variants via NGS, the mutations detected in the proband and their parents were confirmed by Sanger sequencing. Detected variants were annotated using major genetic databases, including Online Mendelian Inheritance in Man (OMIM; https://www.ncbi.nlm.nih.gov/omim), the Human Gene Mutation Database (HGMD; https://www.hgmd.org), and ClinVar, to determine their known disease associations. All identified mutations were classified according to the American College of Medical Genetics and Genomics (ACMG) guidelines for the interpretation of sequence variants (10). The Rare Exome Variant Ensemble Learner (REVEL) score was employed to predict the pathogenicity of missense variants.

### 2.4 Statistical analysis

Categorical data were presented as frequencies (n) and percentages (%), while continuous variables were expressed as mean ± standard deviation (M ± SD). For between-group comparisons, the Mann–Whitney U test and one-way analysis of covariance (ANCOVA) were employed for continuous variables, while Fisher's exact test was used for comparing categorical variables (rates or proportions). *p* < 0.05 was considered statistically significant. All statistical analyses were performed using IBM SPSS Statistics version 29.0 (IBM Corp., New York, NY, USA), and graphs were generated using GraphPad Prism version 10.6 (GraphPad Software, San Diego, CA, USA).

## 3 Results

### 3.1 Demographic and clinical characteristics of children with NTCPD

The patients were sequentially assigned identifiers P01 to P19 according to their enrollment sequence including 13 males and 6 females (male-to-female ratio = 2.2:1). Patient ages ranged from 1 month 29 days to 4 years 7 months, with a median age of 11.4 months. All enrolled children were unrelated and exhibited normal growth parameters; furthermore, none presented with pruritus, abnormal stool color or hepatosplenomegaly, nor did they have abnormalities in their birth, family and past medical histories. Among them, one case (P04) exhibited elevated direct bilirubin, one (P19) showed elevated levels of both direct and indirect bilirubin, and one (P08) had a total serum bilirubin level approaching the upper limit of normal. The remaining patients displayed varying degrees of indirect hyperbilirubinemia. Additionally, cases P02 and P19 had markedly elevated liver enzymes, while no significant elevation was observed in the remaining children, including all in the non-jaundice group (Table 1).

**Table 1:** Demographic and clinical characteristics of children with NTCPD.

Case Number	Gender	Age (month)	C.C.	BA* (μmol/L)	ALT (U/L)	AST (U/L)	TB (μmol/L)	DB (μmol/L)	IB (μmol/L)	γ-GT (U/L)
P01	Male	1	Jaundice	147.7	23.8	26.4	58.9*	4.9	54.0*	142.0*
P02	Male	2	Jaundice	139.5	180.0*	194.0*	219.5*	7.9	211.6*	257.9*
P03	Female	1	Jaundice	261.5	30.8	36.4	64.3*	6.7	57.6*	78.7*
P04	Male	1	Jaundice	99.7	10.7	33.2	56.2*	52.0*	4.7	118.1*
P05	Female	5	IH	197.9	25.6	43.7	7.2	2.7	4.5	67.0
P06	Male	2	Jaundice	167.0	39.0	40.0	102.4*	7.8	94.6*	115.7*
P07	Male	2	Jaundice	120.1	25.0	32.1	71.4*	3.6	67.8*	95.6*
P08	Female	1	Jaundice	82.8	55.5	75.7	19.8	3.8	16.0	42.0*
P09	Male	4	IH	309.4	44.4	46.2	5.6	1.4	4.2	24.5
P10	Male	2	Jaundice	147.0	36.5	41.4	40.3*	4.1	36.2*	98.0*
P11	Male	4	IH	119.0	28.0	34.2	7.7	2.8	4.9	14.3
P12	Male	6	IH	153.0	20.0	38.0	4.9	1.3	3.6	14.0
P13	Male	55	IH	149.0	18.8	38.7	10.4	2.4	8.0	10.1
P14	Female	19	IH	229.3	16.1	37.9	4.5	1.1	3.4	9.4
P15	Female	4	IH	200.0	34.0	43.7	7.8	2.1	5.7	46.7*
P16	Male	52	IH	344.0	21.7	38.6	15.0	4.4	10.6	14.4
P17	Male	18	IH	674.5	23.0	34.0	13.2	2.1	11.1	14.0
P18	Female	3	IH	341.7	27.0	37.0	4.9	3.0	1.9	17.0
P19	Male	2	Jaundice	188.7	122.0*	167.0*	139.0*	81.0*	58.0*	198.5*

Reference intervals are as follows: ALT:7–40 U/L, AST:14–44 U/L, BA: 0–10.0 μmol/L, TB <26 μmol/L, DB: 0–10 μmol/L, IB <26 μmol/L, γ-GT: 5–19 U/L.

* The asterisk symbol (*) marks the indices that were significantly outside the normal range. C.C, chief complaint; IH, incidental hypercholanemia.

### 3.2 Comparison of liver function indicators between jaundice and IH group

Patients in the jaundice group were significantly younger than those in the IH group, as indicated by a *p*-value of 0.003. After adjusting for age, the results demonstrated that although various patterns of bilirubin elevation were observed in the jaundice group, indirect bilirubin elevation remained predominant compared to the IH group (*p* = 0.012). Furthermore, *γ*-glutamyl transferase (*γ*-GT) levels were significantly higher in the jaundice group (*p* = 0.001), while no statistically significant differences were observed in total bile acids (TBA) and liver enzymes between the two groups (*p* > 0.05) (Table 2).

**Table 2:** Comparison of liver function indicators between jaundice and IH group.

Indicator	IH Group (*N* = 10)	Jaundice Group (*N* = 9)	F/*χ*^2^	*P*
Gender(Male, %）	6（60.0%）	7（77.8%）	/	0.628
Age(Month)[IQR (M ± SD)	5.5 (16.7 ± 20.4)	2.0 (1.8 ± 0.9)	/	0.003*
BA(μmol/L)[IQR (M ± SD)	214.7 (271.8 ± 162.8)	147.0 (150.4 ± 52.8)	2.919	0.107
ALT(U/L)[IQR (M ± SD)	24.3 (25.9 ± 8.3)	36.5 (58.1 ± 56.0)	1.978	0.179
AST(U/L)[IQR (M ± SD)	38.3 (39.2 ± 4.1)	40.0 (71.8 ± 63.6)	1.866	0.191
TB(μmol/L)[IQR (M ± SD)	7.5 (8.1 ± 3.6)	64.3 (85.8 ± 60.9)	12.689	0.003*
DB(μmol/L)[IQR (M ± SD)	2.3 (2.3 ± 1.0)	6.7 (19.1 ± 27.9)	2.743	0.117
IB(μmol/L)[IQR (M ± SD)	4.7 (5.8 ± 3.1)	57.6 (66.7 ± 60.7)	7.944	0.012*
γ-GT(U/L)[IQR (M ± SD)	14.4 (23.1 ± 18.9)	115.7 (127.4 ± 65.3)	15.758	0.001*

IH, incidental hypercholanemia.

* *p* < 0.05.

### 3.3 SLC10A1 genotyping results

All patients were found to carry *SLC10A1* gene mutations and carried the pathogenic pS267F mutation. Among them, 14 cases had homozygous mutations, specifically c.800C > T: p.Ser267Phe (chr14:70245193; NM_003049.4:exon4). The remaining 5 cases exhibited compound heterozygous mutations, including c.263T > C: p.Ile88Thr, c.101T > C: p.Met34Thr, c.665T > C: p.Leu222Ser, c.551delT: p.Met184Serfs*9, c.896T > C: p.Leu299Pro, and c.654_674dup: p.Thr219_Thr225dup.

Specifically, patient P02 carried mutations at two distinct sites (c.800C > T and c.263T > C); patient P03 presented with compound heterozygous mutations at three distinct sites (c.800C > T, c.101T > C and c.665T > C); patient P05 exhibited a variant involving a microdeletion (c.800C > T and c.551delT); patient P17 carried mutations at two distinct sites (c.800C > T and c.896T > C) and patient P18 presented with variants comprising an in-frame duplication (c.800C > T and c.654_674dup). Four novel mutations were identified in this cohort: c.101T > C (P03), c.551delT (P05), c.896T > C (P17), and c.654_674dup (P18) (Table 3). According to ACMG guidelines, c.101T > C (PM2 + BP4), c.896T > C (PM2), and c.654_674dup (PM2 + PM4) were classified as variants of uncertain significance, whereas c.551delT (PVS1 + PM2 + PM3 + PP4) was assessed as likely pathogenic. REVEL functional predictions indicated that c.101T > C was benign, while the predictions for c.896T > C variant was inconclusive.

**Table 3:** The mutations in the *SLC10A1* gene of 19 children with NTCPD.

Patients	SLC10A1 genotypes			Effects
	Patient (Het/Homo)	Father (Het/Homo)	Mother (Het/Homo)	
P01	c.800C > T(hom)	c.800C > T(het)	c.800C > T(het)	p.S267F
P02	c.800C > T(het) and c.263T > C（het）	c.800C > T(het)	c.263T > C(het)	p.S267F and p.I88T
P03	c.800C > T(het) and c.101T > C(het)^a^ and c.665T > C (het)	c.101T > C(het) and c.665T > C(het)	c.800C > T(het)	p.S267F and p.M34T and p.L222S
P04	c.800C > T(hom)	c.800C > T(het)	c.800C > T(het)	p.S267F
P05	c.800C > T(het) and c.551delT^a^	c.551delT(het)	c.800C > T(het)	p.S267F and p.M184Sfs^a^9
P06	c.800C > T(hom)	c.800C > T(het)	c.800C > T(het)	p.S267F
P07	c.800C > T(hom)	c.800C > T(het)	c.800C > T(het)	p.S267F
P08	c.800C > T(hom)	c.800C > T(het)	c.800C > T(het)	p.S267F
P09	c.800C > T(hom)	c.800C > T(het)	c.800C > T(het)	p.S267F
P10	c.800C > T(hom)	c.800C > T(het)	c.800C > T(het)	p.S267F
P11	c.801C > T(hom)	c.801C > T(het)	c.801C > T(het)	p.S267F
P12	c.802C > T(hom)	c.802C > T(het)	c.802C > T(het)	p.S267F
P13	c.803C > T(hom)	c.803C > T(het)	c.803C > T(het)	p.S267F
P14	c.804C > T(hom)	c.804C > T(het)	c.804C > T(het)	p.S267F
P15	c.805C > T(hom)	c.805C > T(het)	c.805C > T(het)	p.S267F
P16	c.806C > T(hom)	c.806C > T(het)	c.806C > T(het)	p.S267F
P17	c.800C > T(het) and c.896T > C(het)^a^	c.800C > T(het)	c.896T > C(het)	p.S267F and p.L299P
P18	c.800C > T(het) and c.654_674dup(het)^a^	c.800C > T(het)	c.654_674dup(het)	p.S267F and p.Thr219_Thr225dup
P19	c.806C > T(hom)	c.806C > T(het)	c.806C > T(het)	p.S267F

Het, heterozygous; Homo, homozygous.

a New mutation type.

### 3.4 Longitudinal changes in key liver function indicators in children with NTCPD

**Figure 1:**
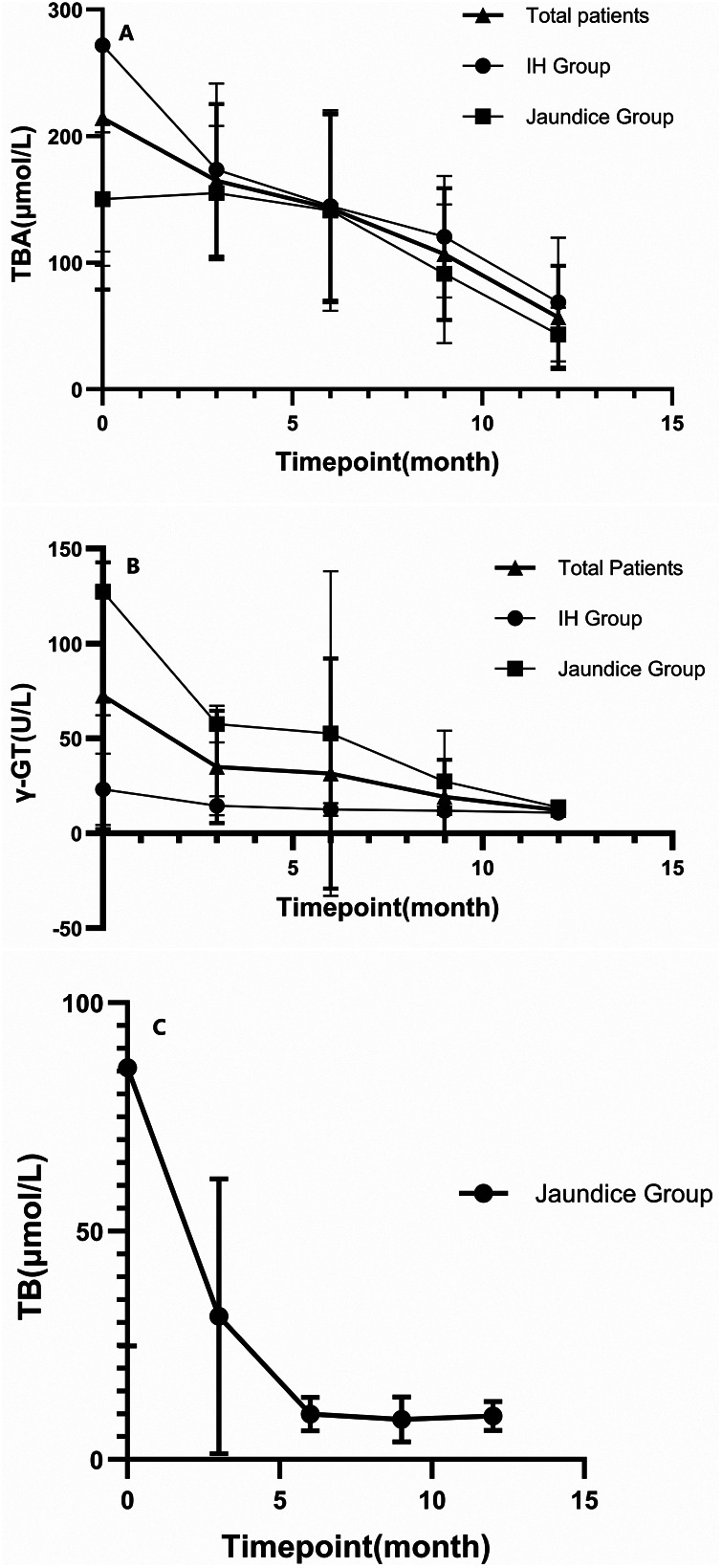
Longitudinal changes in key liver function indicators in children with NTCPD. **A**: Changes in TBA over time. **B**: Changes in *γ*-GT over time. **C**: Changes in total bilirubin in children with jaundice over time. IH, incidental hypercholanemia.

All patients completed the 12-month follow-up period. Bilirubin levels in jaundiced children returned to normal by the 6th month. The abnormal *γ*-GT and liver enzyme levels in all children also normalized by month 9. Both bilirubin levels and *γ*-GT levels remained stable throughout the subsequent follow-up period, with no observed abnormal elevations. Although mild fluctuations in BA levels were noted in individual cases during follow-up, an overall declining trend was observed (Figure 1).

## 4 Discussion

We report on 19 pediatric cases of NTCPD to provide additional insights into this disorder. In our cohort, the median age was 11.4 months, with a male predominance. As NTCPD is an autosomal recessive disorder, the expected sex ratio is theoretically 1:1. However, in our patient cohort, the observed male-to-female ratio was 2.2:1. This discrepancy is most likely attributable to the limited sample size and the inherent referral bias of a single-center retrospective study, as well as the potential influences from other genetic or environmental modifiers that are not yet fully understood. The clinical presentations were heterogeneous. Younger children primarily presented with jaundice as the chief complaint. Previous studies have documented additional reasons for medical consultation, such as dark urine, elevated liver enzymes, abnormal liver function tests during non-hepatic infections (including pneumonia or tonsillitis), and findings observed during routine child health examinations. Some affected children have also been reported to exhibit accompanying conditions including growth retardation, feeding difficulties, cholelithiasis, vitamin D deficiency, and potential neurological impairment (8, 9). However, in our study, no cases of growth retardation or vitamin D deficiency were observed.

NTCPD is characterized by refractory hypercholanemia as its prominent manifestation. During the neonatal and infantile periods, when the BA metabolic system is still immature, the impact of NTCPD may be more pronounced. In our cohort, analysis revealed significant differences in bilirubin between two groups. However, after adjusting for age no statistically significant difference in BA levels was found. These findings suggest the potential involvement of alternative pathways in BA metabolism. Indeed, studies have identified compensatory BA transporters beyond NTCP. Specifically, the organic anion-transporting polypeptide heterodimer OATP1B1/1B3, located on the basolateral membrane of hepatocytes, facilitates sodium-independent uptake of BAs and bilirubin (11). Additionally, other mechanisms such as the heterodimeric OST*α*/OST*β* expressed on hepatocyte membranes, bifunctional microsomal epoxide hydrolase (mEH), and bile acid sulfation play a role in bile acid homeostasis (12–14).

Previous case series have reported highly variable peak BA levels, ranging from 40 to 1531*μ*mol/L. For instance, Schneider et al. (9). described BA fluctuations between 131 and 700μmol/L among their cases, with a sharp transient peak reaching 1500μmol/L during follow-up. This peak coincided with an episode of viral gastroenteritis. In contrast, in our study, BA levels demonstrated limited variability across individual patients at the same time point. Within each patient, BA showed a consistent downward trend over time without substantial fluctuations. The precise mechanisms underlying these pronounced BA surges remain unclear. It is hypothesized that interruptions in the enterohepatic circulation—potentially, secondary to inflammation caused by conditions such as viral gastroenteritis—could lead to either increased bile acid synthesis or reduced hepatocellular uptake via non-NTCP-mediated pathways. However, further data are needed to clarify the exact triggers of these BA fluctuations. Nevertheless, throughout the follow-up period, BA levels in patients followed an overall downward trend without uncontrolled escalation; additionally, a subset of children demonstrated a gradual normalization of serum BA concentrations (15).

Our study found that *γ*-GT levels were significantly higher in children presenting with jaundice than those in the IH group. This observation may be primarily associated with dysregulation of BA metabolism resulting from NTCPD and the consequent reactive changes in the biliary system. Dong et al. (16) reported histopathological findings in 11 children with NTCPD, which included mildly increased cytoplasmic lipid droplets, portal inflammation, and mild cholestasis in some cases. *γ*-GT is predominantly located in the canalicular membrane of hepatocytes and the epithelium of the bile ducts. Elevated serum *γ*-GT levels typically occur when the biliary system is affected by cholestasis, inflammation, or epithelial injury. In jaundiced children with NTCPD, the marked elevation in *γ*-GT indicates reactive alterations in the biliary system, which may encompass stress responses and hyperplasia of small bile duct epithelial cells, or subtle adaptive modifications in biliary architecture. Therefore, *γ*-GT may serve as a useful indicator for distinguishing different clinical phenotypes of NTCPD; however, its utility requires validation in larger, prospective cohorts with systematic documentation of feeding practices (17).

Ho et al. (18) revealed an ethnic-specific distribution of *SLC10A1* polymorphic variants. The c.800C > T mutation was exclusively identified in Chinese Americans, with a minor allele frequency (MAF) of 7.5%, but was absent in African American, European American, and Hispanic populations. In contrast, the c.668T > C variant had an MAF of 5.5% in African Americans and was rarely observed in Hispanics. Additionally, single cases of c.836T > C and c.940A > G were detected in Chinese Americans and Hispanic Americans, respectively. Pan et al. (19) reported allele frequencies for the c.800C > T variant as 7.4% (23/312) in Chinese, 3.1% (9/294) in Korean, and 9.2% (28/306) in Vietnamese populations.Consistent with these previous findings, all patients in our cohort were of Chinese origin, which explains the notably higher frequency of c.800C > T compared to other variants in our study. The remarkably high allele frequency of c.800C > T may be explained by potential selective advantages (20, 21).

The c.800C > T variant results in an almost complete loss (>98%) of transport activity for BAs such as taurocholic acid and cholic acid, while showing no impact on the transport of non-bile acid substrates like estrogen sulfate. This indicates that the Ser267 residue forms part of a specific recognition domain essential for bile acid binding (18). Previous clinical reports suggest that in the presence of precipitating factors such as prematurity and hyperbilirubinemia, some carriers of pathogenic *SLC10A1* variants may exhibit biochemical features of NTCPD, including hypercholanemia and early infantile cholestasis (22). In their analysis of prior cases, Schneider et al. (9) in their analysis of prior cases, observed that children homozygous for the c.800C > T mutation generally demonstrated lower BA levels.In our cohort, patients with compound heterozygous mutations (e.g., P17, P18) presented with relatively higher BA levels. This phenotypic variation is likely influenced by the residual transport function of NTCP and the corresponding regulatory effects on BA synthesis. However, conclusions regarding a clear genotype-phenotype correlation remain preliminary and require longer-term observation and more comprehensive data analysis for validation.

The study is limited by its single-center design and sample size, which may affect the statistical power for correlating rare genotypes with phenotypes. Furthermore, the young age of our cohort and the relatively short follow-up period of one year limit the conclusions that can be drawn regarding long-term growth parameters and developmental outcomes. Future studies with extended follow-up are warranted to assess these longer-term effects. Additionally, the interpretation of BA fluctuation mechanisms relies largely on inferences from the literature; and lacks of functional experimental validation from this cohort. Future studies should involve multi-center, large-scale collaborations to establish a comprehensive genotype-phenotype spectrum and utilize *in vitro* cell models to validate the functional impact of the newly identified mutations.

## Data Availability

The datasets presented in this study can be found in online repositories. The names of the repository/repositories and accession number(s) can be found in the article/Supplementary Material.
