# Supplementary material for: Clinical and genetic characteristics of children with sodium taurocholate cotransporting poly-peptide deficiency
Source: Front Pediatr. 2026 Jun 10;14:1784165. doi: 10.3389/fped.2026.1784165 (PMC13290667; doi:10.3389/fped.2026.1784165)
Supplement: Supplementary file 1 [file Datasheet1.pdf]

M030V2：代谢性肝病基因检测 panel V6 基因-疾病列表

相对旧项目 M019V2：代谢性肝病基因检测 panel-V2，新增 40 个基因，共 611 个基因。

panel 疾病大类	基因列表	疾病小类	新增
氨基酸代谢缺陷	AASS	高赖氨酸血症（OMIM:238700）	否
氨基酸代谢缺陷	ACAT1	β-酮硫酶缺乏症（OMIM:203750）	否
氨基酸代谢缺陷	ADK	腺苷激酶缺乏性高甲硫氨酸血症（OMIM:614300）	否
氨基酸代谢缺陷	AHCY	S-腺苷高半胱氨酸水解酶缺乏性高甲硫氨酸血症（OMIM:613752）	否
氨基酸代谢缺陷	AMT	甘氨酸脑病（OMIM:605899）	否
氨基酸代谢缺陷	BCKDHA	枫糖尿病 2 型/1A 型/1B 型（OMIM:248600）	否
氨基酸代谢缺陷	BCKDHB	枫糖尿病 2 型/1A 型/1B 型（OMIM:248600）	否
氨基酸代谢缺陷	CBS	胱硫醚β合成酶缺乏性高胱氨酸尿症（OMIM:236200）	否
氨基酸代谢缺陷	DBT	枫糖尿病 2 型/1A 型/1B 型（OMIM:248600）	否
氨基酸代谢缺陷	DDC	左旋芳香族氨基酸脱羧酶缺乏症（OMIM:608643）	否
氨基酸代谢缺陷	DLD	枫糖尿病 3 型（OMIM:246900）	否
氨基酸代谢缺陷	FAH	酪氨酸血症 1 型（OMIM:276700）	否
氨基酸代谢缺陷	GCH1	BH4 缺乏性高苯丙氨酸血症 B 型（OMIM:233910）	否
氨基酸代谢缺陷	GCSH	甘氨酸脑病（OMIM:605899）	否
氨基酸代谢缺陷	GLDC	甘氨酸脑病（OMIM:605899）	否
氨基酸代谢缺陷	GNMT	甘氨酸 N-甲基转移酶（OMIM:606664）	否
氨基酸代谢缺陷	GSTZ1	酪氨酸血症 Ib 型	否
氨基酸代谢缺陷	HGD	黑尿酸尿症（OMIM:203500）	否
氨基酸代谢缺陷	HMGCL	3-羟基-3-甲基戊二酰辅酶 A 裂解酶缺乏症（OMIM:246450）	否
氨基酸代谢缺陷	HPD	酪氨酸血症 3 型（OMIM:276710）	否
氨基酸代谢缺陷	MAT1A	蛋氨酸腺苷转移酶 I / III 缺乏导致的高蛋氨酸血症（OMIM:250850）	否
氨基酸代谢缺陷	NFE2L2	免疫缺陷-发育迟滞-高同型半胱氨酸血症（OMIM:617744）	否
氨基酸代谢缺陷	PAH	苯丙酮尿症（OMIM:261600）	否
氨基酸代谢缺陷	PRODH	高脯胺酸血症 1 型（OMIM:239500）	否
氨基酸代谢缺陷	PTS	BH4 缺乏性高苯丙氨酸血症 A 型（OMIM:261640）	否
氨基酸代谢缺陷	SLC25A13	希特林蛋白缺乏症	否
氨基酸代谢缺陷	SLC25A15	高鸟氨酸血症-高氨血症-高同型瓜氨酸尿综合征（OMIM:238970）	否
氨基酸代谢缺陷	TAT	酪氨酸血症 2 型（OMIM:276600）	否
氨基酸代谢缺陷	UROC1	尿刊酸酶缺乏症（OMIM:276880）	否
卟啉病相关	ALAD	急性肝卟啉病（OMIM:612740）	否
卟啉病相关	ALAS2	X 连锁原卟啉病（OMIM:300752）	否
卟啉病相关	CPOX	遗传性粪卟啉病（OMIM:121300）	否
卟啉病相关	FECH	红细胞生成性原卟啉病（OMIM:177000）	否
卟啉病相关	HMBS	急性间歇性卟啉病（OMIM:176000）	否
卟啉病相关	UROD	肝红细胞生成型血卟啉病（OMIM:176100）	否

卟啉病相关	UROS	先天性红细胞生成性卟啉病（OMIM:263700）	否
胆红素代谢障碍	ABCC2	Dubin-Johnson 综合征（OMIM:237500）	否
胆红素代谢障碍	SLCO1B1	Rotor 综合征（OMIM:237450）	否
胆红素代谢障碍	SLCO1B3	Rotor 综合征（OMIM:237450）	否
胆红素代谢障碍	TRMU	小儿暂时性肝功能衰竭综合征（OMIM:613070）	否
胆红素代谢障碍	UGT1A1	家族性暂时性新生儿高胆红素血症（OMIM:237900）	否
胆汁淤积相关	ABCB11	进行性家族性肝内胆汁淤积症 2 型（OMIM:601847）	否
胆汁淤积相关	ABCB4	进行性家族性肝内胆汁淤积症 3 型（OMIM:602347）	否
胆汁淤积相关	ABCD3	先天性胆汁酸合成障碍 5 型（OMIM:616278）	否
胆汁淤积相关	ACOX2	先天性胆汁酸合成缺陷 6 型（OMIM:617308）	否
胆汁淤积相关	AKR1D1	先天性胆汁酸合成障碍 2 型（OMIM:235555）	否
胆汁淤积相关	AMACR	先天性胆汁酸合成障碍 4 型（OMIM:214950）	否
胆汁淤积相关	APCS	淀粉样变性易感性	否
胆汁淤积相关	ATP8B1	进行性家族性肝内胆汁淤积症 1 型（OMIM:211600）	否
胆汁淤积相关	BAAT	家族性超胆烷（OMIM:607748）	否
胆汁淤积相关	BLVRA	高胆绿素血症（OMIM:614156）	否
胆汁淤积相关	CLDN1	新生儿鱼鳞病-硬化性胆管炎综合征（OMIM:607626）	否
胆汁淤积相关	CYP7B1	先天性胆汁酸合成障碍 3 型（OMIM:613812）	否
胆汁淤积相关	EPHX1	家族性超胆烷（OMIM:607748）	否
胆汁淤积相关	FGA	家族性内脏淀粉样病变（OMIM:105200）	否
胆汁淤积相关	GSN	芬兰型淀粉样变性（OMIM:105120）	否
胆汁淤积相关	HSD3B7	先天性胆汁酸合成障碍 1 型（OMIM:607765）	否
胆汁淤积相关	IL31RA	原发性局限性皮肤淀粉样变性病 2 型（OMIM:613955）	否
胆汁淤积相关	JAG1	Alagille 综合征 1 型（OMIM:118450）	否
胆汁淤积相关	LYZ	家族性内脏淀粉样病变（OMIM:105200）	否
胆汁淤积相关	MARS1	间质性肺病及肝病（OMIM:615486）	否
胆汁淤积相关	MYO5B	腹泻伴微绒毛萎缩 2 型（OMIM:251850）	否
胆汁淤积相关	NOTCH2	Alagille 综合征 2 型（OMIM:610205）	否
胆汁淤积相关	NR1H4	进行性家族性肝内胆汁淤积 5 型（OMIM:617049）	否
胆汁淤积相关	OSMR	原发性局限性皮肤淀粉样变性病 1 型（OMIM:105250）	否
胆汁淤积相关	SLC10A2	初级胆汁酸吸收障碍（OMIM:613291）	否
胆汁淤积相关	SLC27A5	胆汁酸-CoA 连接酶缺陷（PMID: 22089923）	否
胆汁淤积相关	TJP2	进行性家族性肝内胆汁淤积症 4 型（OMIM:615878）	否
胆汁淤积相关	TTR	家族性转甲状腺素蛋白淀粉样变性病（OMIM:105210）	否
胆汁淤积相关	USP53	γ-谷氨酰转移酶（GGT）胆汁淤积症（OMIM:）	是
胆汁淤积相关	UTP4	北美印第安儿童肝硬化（OMIM:604901）	否
胆汁淤积相关	VIL1	进行性微管胆汁淤积	否
胆汁淤积相关	VIPAS39	关节挛缩、肾功能不全及胆汁淤积综合征 2 型（OMIM:613404）	否
胆汁淤积相关	VPS33B	关节挛缩、肾功能不全及胆汁淤积综合征 1 型（OMIM:208085）	否
短肋胸廓发育不良	DYNC2H1	短肋胸廓发育不良伴或不伴多指综合征 3 型（OMIM:613091）	否
短肋胸廓发育不良	DYNC2I1	短肋胸廓发育不良伴或不伴多指综合征 8 型（OMIM:615503）	否
短肋胸廓发育不良	DYNC2I2	短肋胸廓发育不良伴或不伴多指综合征 11 型（OMIM:615633）	否

短肋胸廓发育不良	DYNLT2B	短肋胸廓发育不良 17 型伴或不伴有畸形（OMIM:617405）	否
短肋胸廓发育不良	IFT140	短肋胸廓发育不良伴或不伴多指综合征 9 型（OMIM:266920）	否
短肋胸廓发育不良	IFT172	短肋胸廓发育不良伴或不伴多指综合征 10 型（OMIM:615630）	否
短肋胸廓发育不良	IFT80	短肋胸廓发育不良伴或不伴多指综合征 2 型（OMIM:611263）	否
短肋胸廓发育不良	KIAA0586	短肋胸廓发育不良伴多指综合征 14 型（OMIM:616546）	否
短肋胸廓发育不良	NEK1	短肋胸廓发育不良伴或不伴多指综合征 6 型（OMIM:263520）	否
短肋胸廓发育不良	WDR19	短肋胸廓发育不良伴或不伴多指综合征 5 型（OMIM:614376）	否
短肋胸廓发育不良	WDR35	短肋胸廓发育不良伴或不伴多指综合征 7 型（OMIM:614091）	否
多囊肝病	DNAJB11	多囊肾 6 型伴或不伴多囊肝（OMIM:618061）	是
多囊肝病	LRP5	多囊肝病 4 型伴或不伴肾囊肿（OMIM:617875）	是
肝癌	AXIN1	肝细胞癌（OMIM:114550）	是
肝癌	CASP8	肝细胞癌（OMIM:114550）	是
肝癌	IGF2R	肝细胞癌（OMIM:114550）	是
肝癌	MET	体细胞幼儿肝细胞癌（OMIM:114550）	是
肝癌	PDGFRL	肝细胞癌（OMIM:114550）	是
肝癌	TP53	肝细胞癌（OMIM:114550）	是
金属代谢缺陷	ATP7B	肝豆状核变性（OMIM:277900）	否
金属代谢缺陷	CP	无铜蓝蛋白血症（OMIM:604290）	否
金属代谢缺陷	FTH1	遗传性血色病 5 型（OMIM:615517）	否
金属代谢缺陷	HAMP	遗传性血色病 2B 型（OMIM:613313）	否
金属代谢缺陷	HFE	遗传性血色病 1 型（OMIM:235200）	否
金属代谢缺陷	HJV	遗传性血色病 2A 型（OMIM:602390）	否
金属代谢缺陷	SLC11A2	小细胞低色素性贫血伴铁超负荷 1 型（OMIM:206100）	否
金属代谢缺陷	SLC30A10	高锰血症伴肌张力失调 1 型（OMIM:613280）	否
金属代谢缺陷	SLC40A1	遗传性血色病 4 型（OMIM:606069）	否
金属代谢缺陷	TF	无转铁蛋白血症（OMIM:209300）	否
金属代谢缺陷	TFR2	遗传性血色病 3 型（OMIM:604250）	否
酶类缺陷	AARS2	复合型氧化磷酸化缺陷症 8 型（OMIM:614096）	否
酶类缺陷	ACADS	短链酰基辅酶 A 脱氢酶缺乏症（OMIM:201470）	否
酶类缺陷	ACOX1	过氧化物酶酰基辅酶 A 氧化酶缺乏症（OMIM:264470）	否
酶类缺陷	ADA	腺苷脱氨酶缺乏症（OMIM:102700）	否
酶类缺陷	AIFM1	复合型氧化磷酸化缺陷症 6 型（OMIM:300816）	否
酶类缺陷	ALPL	儿童型低磷酸酯酶症（OMIM:241510）	否
酶类缺陷	ATP5F1A	复合型氧化磷酸化缺陷症 22 型（OMIM:616045）	否
酶类缺陷	BTD	生物素酶缺乏症（OMIM:253260）	否
酶类缺陷	C1QBP	联合氧化磷酸化缺陷 33 型(OMIM:617713)	否
酶类缺陷	CARS2	复合型氧化磷酸化缺陷症 27 型（OMIM:616672）	否
酶类缺陷	CAT	过氧化氢酶缺乏症（OMIM:614097）	否
酶类缺陷	COQ2	原发性辅酶 Q10 缺乏症 1 型（OMIM:607426）	否
酶类缺陷	DHFR	二氢叶酸还原酶缺乏性巨幼细胞贫血（OMIM:613839）	否
酶类缺陷	DPYS	二氢嘧啶酶缺乏症（OMIM:222748）	否
酶类缺陷	EARS2	复合型氧化磷酸化缺陷症 12 型（OMIM:614924）	否

酶类缺陷	ELAC2	复合型氧化磷酸化缺陷症 17 型（OMIM:615440）	否
酶类缺陷	FARS2	复合型氧化磷酸化缺陷症 14 型（OMIM:614946）	否
酶类缺陷	G6PD	葡萄糖-6-磷酸脱氢酶缺乏症（OMIM:300908）	否
酶类缺陷	GFM1	复合型氧化磷酸化缺陷症 1 型（OMIM:609060）	否
酶类缺陷	HADH	3-羟酰基辅酶 A 脱氢酶缺乏（OMIM:231530）	否
酶类缺陷	HSD17B4	过氧化物酶体 D-双功能蛋白缺乏症（OMIM:261515）	否
酶类缺陷	LDHB	乳酸脱氢酶 B 缺乏症（OMIM:614128）	否
酶类缺陷	LYRM4	复合型氧化磷酸化缺陷症 19 型（OMIM:615595）	否
酶类缺陷	MRPL3	复合型氧化磷酸化缺陷症 9 型（OMIM:614582）	否
酶类缺陷	MRPL44	复合型氧化磷酸化缺陷症 16 型（OMIM:615395）	否
酶类缺陷	MRPS16	复合型氧化磷酸化缺陷症 2 型（OMIM:610498）	否
酶类缺陷	MRPS22	复合型氧化磷酸化缺陷症 5 型（OMIM:611719）	否
酶类缺陷	MRPS7	复合型氧化磷酸化缺陷症 34 型（OMIM:617872）	否
酶类缺陷	MTFMT	复合型氧化磷酸化缺陷症 15 型（OMIM:614947）	否
酶类缺陷	MTO1	复合型氧化磷酸化缺陷症 10 型（OMIM:614702）	否
酶类缺陷	MTRFR	复合型氧化磷酸化缺陷症 7 型（OMIM:613559）	否
酶类缺陷	NARS2	复合型氧化磷酸化缺陷症 24 型（OMIM:616239）	否
酶类缺陷	PEX1	过氧化物酶体生物合成障碍 1A 型（OMIM:214100）	否
酶类缺陷	PEX10	过氧化物酶体生物合成障碍 6A 型（OMIM:614870）	否
酶类缺陷	PEX11B	过氧化物酶体生物合成障碍 14B 型（OMIM:614920）	否
酶类缺陷	PEX12	过氧化物酶体生物合成障碍 3B 型（OMIM:266510）	否
酶类缺陷	PEX13	过氧化物酶体生物合成障碍 11A 型（OMIM:614883）	否
酶类缺陷	PEX14	过氧化物酶体生物合成障碍 13A 型（OMIM:614887）	否
酶类缺陷	PEX16	过氧化物酶体生物合成障碍 8A 型（OMIM:614876）	否
酶类缺陷	PEX19	过氧化物酶体生物合成障碍 12A 型（OMIM:614886）	否
酶类缺陷	PEX2	过氧化物酶体生物合成障碍 5A 型（OMIM:614866）	否
酶类缺陷	PEX26	过氧化物酶体生物合成障碍 7A 型（OMIM:614872）	否
酶类缺陷	PEX3	过氧化物酶体生物合成障碍 10A 型（OMIM:614882）	否
酶类缺陷	PEX5	过氧化物酶体生物合成障碍 2B 型（OMIM:202370）	否
酶类缺陷	PEX6	过氧化物酶体生物合成障碍 4B 型（OMIM:614863）	否
酶类缺陷	PEX7	过氧化物酶体生物合成障碍 9B 型（OMIM:614879）	否
酶类缺陷	PHKG1	磷酸化酶激酶缺乏症	否
酶类缺陷	PNPT1	复合型氧化磷酸化缺陷症 13 型（OMIM:614932）	否
酶类缺陷	PRDX1	过氧化物还原酶（PMID: 26301632）	否
酶类缺陷	RMND1	复合型氧化磷酸化缺陷症 11 型（OMIM:614922）	否
酶类缺陷	SERPINA1	α1 抗胰蛋白酶缺乏症（OMIM:613490）	否
酶类缺陷	SFXN4	复合型氧化磷酸化缺陷症 18 型（OMIM:615578）	否
酶类缺陷	SI	先天性蔗糖酶-异麦芽糖酶缺乏症（OMIM:222900）	是
酶类缺陷	ST3GAL5	神经节苷脂生物合成酶（PMID: 24026681）	否
酶类缺陷	SUMF1	多发性硫酸脂酶缺乏症（OMIM:272200）	否
酶类缺陷	TARS2	复合型氧化磷酸化缺陷症 21 型（OMIM:615918）	否
酶类缺陷	TRMT10C	复合型氧化磷酸化缺陷症 30 型（OMIM:616974）	否

酶类缺陷	TSFM	复合型氧化磷酸化缺陷症 3 型（OMIM:610505）	否
酶类缺陷	TUFM	复合型氧化磷酸化缺陷症 4 型（OMIM:610678）	否
酶类缺陷	UPB1	β-脲基丙酸酶缺乏症（OMIM:613161）	否
酶类缺陷	VARS2	复合型氧化磷酸化缺陷症 20 型（OMIM:615917）	否
囊性纤维化性肝病	APC	遗传性硬纤维瘤病;DESMD（OMIM:135290）	是
囊性纤维化性肝病	CC2D2A	COACH 综合征（OMIM:216360）	否
囊性纤维化性肝病	CEP41	Joubert 综合征 15 型（OMIM:614464）	否
囊性纤维化性肝病	CFTR	囊性纤维化（OMIM:219700）	否
囊性纤维化性肝病	CSPP1	Joubert 综合征 21 型（OMIM:615636）	否
囊性纤维化性肝病	IFT122	颅骨外胚层发育不良 1 型（OMIM:218330）	否
囊性纤维化性肝病	IFT43	颅骨外胚层发育不良 3 型（OMIM:614099）	否
囊性纤维化性肝病	KRT18	家族性肝硬化（OMIM:215600）	否
囊性纤维化性肝病	KRT8	家族性肝硬化（OMIM:215600）	否
囊性纤维化性肝病	LARS1	小儿肝功能衰竭综合征 1 型（OMIM:615438）	否
囊性纤维化性肝病	MKS1	Meckel 综合征 1 型（OMIM:249000）	否
囊性纤维化性肝病	MUC5B	肺纤维化，特发性，易感性（OMIM:178500）	否
囊性纤维化性肝病	PIBF1	Joubert 综合征 33 型（OMIM:617767）	否
囊性纤维化性肝病	PRKCSH	多囊性肝病 1 型（OMIM:174050）	否
囊性纤维化性肝病	RPGRIP1L	COACH 综合征（OMIM:216361）	否
囊性纤维化性肝病	SEC63	多囊性肝病 2 型（OMIM:617004）	否
囊性纤维化性肝病	SFTPA1	肺纤维化，特发性，易感性（OMIM:178500）	否
囊性纤维化性肝病	SFTPA2	家族性特发性肺纤维化（OMIM:178500）	否
囊性纤维化性肝病	SP110	2.肝静脉梗阻症伴免疫缺陷（OMIM:235550）	否
囊性纤维化性肝病	TERC	端粒相关的骨髓造血功能衰竭和/或肺间质纤维化 2 型（OMIM:614743）	否
囊性纤维化性肝病	TERT	端粒相关的骨髓造血功能衰竭和/或肺间质纤维化 1 型（OMIM:614742）	否
囊性纤维化性肝病	TMEM107	Meckel 综合征 13 型（OMIM:617562）	否
囊性纤维化性肝病	TMEM216	Meckel 综合征 2 型（OMIM:603194）	否
囊性纤维化性肝病	TMEM67	COACH 综合征（OMIM:216360）	否
尿素循环缺陷	ARG1	精氨酸酶缺乏症（OMIM:207800）	否
尿素循环缺陷	ASL	精氨基琥珀酸尿症（OMIM:207900）	否
尿素循环缺陷	ASS1	瓜氨酸血症 1 型（OMIM:215700）	否
尿素循环缺陷	CPS1	氨甲酰磷酸合成酶 I 缺乏症（OMIM:237300）	否
尿素循环缺陷	NAGS	N-乙酰谷氨酸合成酶缺乏症（OMIM:237310）	否
尿素循环缺陷	OTC	鸟氨酸氨甲酰转移酶缺乏症（OMIM:311250）	否
尿素循环缺陷	SLC7A7	赖氨酸尿性蛋白耐受不良（OMIM:222700）	否
溶酶体病相关	AGA	天冬氨酰葡糖胺尿症（OMIM:208400）	否
溶酶体病相关	ARSA	异染性脑白质营养不良（OMIM:250100）	否
溶酶体病相关	ARSB	粘多糖贮积症 6 型（OMIM:253200）	否
溶酶体病相关	CLN3	神经元蜡样脂质褐素沉积病 3 型（OMIM:204200）	否
溶酶体病相关	CLN5	神经元蜡样脂质褐素沉积病 5 型（OMIM:256731）	否

溶酶体病相关	CLN6	神经元蜡样脂质褐素沉积病 4A 型（OMIM:204300）	否
溶酶体病相关	CLN8	神经元蜡样脂质褐素沉积病 8 型（OMIM:600143）	否
溶酶体病相关	CTSD	神经元蜡样脂质褐素沉积病 10 型（OMIM:610127）	否
溶酶体病相关	CTSF	神经元蜡样脂质褐素沉积病 13 型（OMIM:615362）	否
溶酶体病相关	DNAJC5	神经元蜡样脂质褐素沉积病 4B 型（OMIM:162350）	否
溶酶体病相关	GALNS	粘多糖贮积症 4A 型（OMIM:253000）	否
溶酶体病相关	GBA	戈谢病 1 型（OMIM:230800）	否
溶酶体病相关	GLA	Fabry 病（OMIM:301500）	否
溶酶体病相关	GLB1	粘多糖贮积症 4B 型（OMIM:253010）	否
溶酶体病相关	GM2A	AB 变异型 GM2 神经节苷脂贮积症（OMIM:272750）	否
溶酶体病相关	GNE	唾液酸尿症（OMIM:269921）	否
溶酶体病相关	GNPTAB	粘脂质贮积症 3α/3β型（OMIM:252600）	否
溶酶体病相关	GNPTG	粘脂质贮积症 3γ型（OMIM:252605）	否
溶酶体病相关	GRN	神经元蜡样脂质褐素沉积病 11 型（OMIM:614706）	否
溶酶体病相关	GUSB	粘多糖贮积症 7 型（OMIM:253220）	否
溶酶体病相关	HEXB	Sandhoff 病（OMIM:268800）	否
溶酶体病相关	HGSNAT	粘多糖贮积症 3C 型（OMIM:252930）	否
溶酶体病相关	HYAL1	粘多糖贮积症 9 型（OMIM:601492）	否
溶酶体病相关	IDS	粘多糖贮积症 2 型（OMIM:309900）	否
溶酶体病相关	LIPF	溶酶体酸性脂肪酶不足	否
溶酶体病相关	MCOLN1	粘脂质贮积症 4 型（OMIM:252650）	否
溶酶体病相关	MFSD8	神经元蜡样脂质褐素沉积病 7 型（OMIM:610951）	否
溶酶体病相关	NAGLU	粘多糖贮积症 3B 型（OMIM:252920）	否
溶酶体病相关	NEU1	唾液酸沉积症（OMIM:256550）	否
溶酶体病相关	NPC1	尼曼-匹克病 C1 型（OMIM:257220）	否
溶酶体病相关	NPC2	尼曼-匹克病 C2 型（OMIM:607625）	否
溶酶体病相关	PPT1	神经元蜡样脂质褐素沉积病 1 型（OMIM:256730）	否
溶酶体病相关	PSAP	Saposin+C 缺乏性戈谢病（OMIM:610539）	否
溶酶体病相关	SGSH	粘多糖贮积症 3A 型（OMIM:252900）	否
溶酶体病相关	SLC17A5	婴儿型唾液游离酸贮积症（OMIM:269920）	否
溶酶体病相关	SMPD1	1.尼曼-匹克病 A 型（OMIM:257200）	否
溶酶体病相关	TPP1	神经元蜡样脂质褐素沉积病 2 型（OMIM:204500）	否
碳水化合物代谢缺陷	AGL	糖原累积症 III 型（OMIM:232400）	否
碳水化合物代谢缺陷	ALDOA	糖原累积症 XII 型（OMIM:611881）	否
碳水化合物代谢缺陷	ALDOB	遗传性果糖不耐受（OMIM:229600）	否
碳水化合物代谢缺陷	ENO3	糖原累积症 XIII 型（OMIM:612932）	否
碳水化合物代谢缺陷	FBP1	果糖-1,6-二磷酸酶缺乏症（OMIM:229700）	否
碳水化合物代谢缺陷	FUCA1	岩藻糖苷贮积病（OMIM:230000）	否
碳水化合物代谢缺陷	G6PC1	糖原累积症 Ia 型（OMIM:232200）	否
碳水化合物代谢缺陷	GAA	糖原累积症 II 型（OMIM:232300）	否
碳水化合物代谢缺陷	GALE	差向异构酶缺乏性半乳糖血症（OMIM:230350）	否
碳水化合物代谢缺陷	GALK1	半乳糖激酶缺乏症（OMIM:230200）	否

碳水化合物代谢缺陷	GALT	半乳糖血症（OMIM:230400）	否
碳水化合物代谢缺陷	GBE1	糖原累积症 IV 型（OMIM:232500)	否
碳水化合物代谢缺陷	GYG1	糖原累积症 XV 型（OMIM:613507）	否
碳水化合物代谢缺陷	GYS1	肌肉糖原累积症 0 型（OMIM:611556）	否
碳水化合物代谢缺陷	GYS2	肝糖原累积症 0 型（OMIM:240600）	否
碳水化合物代谢缺陷	KHK	特发性果糖尿症（OMIM:229800）	否
碳水化合物代谢缺陷	LAMP2	Danon 病（OMIM:300257）	否
碳水化合物代谢缺陷	LDHA	糖原累积症 XI 型（OMIM:612933）	否
碳水化合物代谢缺陷	MAN2B1	α-甘露糖苷贮积症（OMIM:248500）	否
碳水化合物代谢缺陷	MANBA	β-甘露糖苷贮积症（OMIM:248510）	否
碳水化合物代谢缺陷	PCK1	细胞内磷酸烯醇式丙酮酸羧激酶缺乏症（OMIM:261680）	否
碳水化合物代谢缺陷	PFKM	糖原累积症 VII 型（OMIM:232800）	否
碳水化合物代谢缺陷	PGAM2	糖原累积症 X 型（OMIM:261670）	否
碳水化合物代谢缺陷	PGM1	糖原累积症 XIV 型（OMIM:614921）	否
碳水化合物代谢缺陷	PHKA1	肌糖原累积症 IXd 型（OMIM:300559）	否
碳水化合物代谢缺陷	PHKA2	糖原累积症 IXa1 型（OMIM:306000）	否
碳水化合物代谢缺陷	PHKB	糖原累积症 IXb 型（OMIM:261750）	否
碳水化合物代谢缺陷	PHKG2	糖原累积症 IXc 型（OMIM:613027）	否
碳水化合物代谢缺陷	PKLR	丙酮酸激酶活性增高症（OMIM:102900）	否
碳水化合物代谢缺陷	PRKAG2	致死性先天性心糖原累积病（OMIM:261740）	否
碳水化合物代谢缺陷	PYGL	糖原累积症 VI 型（OMIM:232700）	否
碳水化合物代谢缺陷	PYGM	糖原累积症 V 型（OMIM:232600）	否
碳水化合物代谢缺陷	SLC37A4	糖原累积症 Ic 型（OMIM:232240）	否
碳水化合物代谢缺陷	TALDO1	转醛醇酶缺乏症（OMIM:606003）	否
碳水化合物代谢障碍	FBP2	-	是
碳水化合物代谢障碍	LCT	乳糖不耐受症（OMIM:223000）	是
碳水化合物代谢障碍	PDHX	丙酮酸脱氢酶复合物 E3 结合蛋白缺乏症（OMIM:245349）	是
碳水化合物代谢障碍	PRKAG3	骨骼肌糖原含量与代谢 QTL（OMIM: 619030）	是
碳水化合物代谢障碍	SLC17A3	血清尿酸浓度数量性状位点 4；UAQTL4（OMIM:612671）	是
碳水化合物代谢障碍	SLC5A1	葡萄糖-半乳糖吸收不良（OMIM:606824）	是
糖尿病相关	AKT2	非胰岛素依赖性糖尿病（OMIM:125853）	否
糖尿病相关	EIF2AK3	多发性骨骺发育不良伴早发糖尿病（OMIM:226980）	否
糖尿病相关	GLIS3	新生儿糖尿病伴先天性甲状腺功能减退（OMIM:610199）	否
糖尿病相关	HNF1B	非胰岛素依赖性糖尿病（OMIM:125853）	否
糖尿病相关	HNF4A	非胰岛素依赖性糖尿病（OMIM:125853）	否
糖尿病相关	IER3IP1	小头畸形、癫痫和糖尿病综合征（OMIM:614231）	否
糖尿病相关	IL2RA	胰岛素依赖性糖尿病 10 型（OMIM:601942）	否
糖尿病相关	PPARG	非胰岛素依赖性糖尿病（OMIM:125853）	否
糖尿病相关	RFX6	Mitchell-Riley 综合征（OMIM:615710）	否
糖尿病相关	SLC2A2	非胰岛素依赖性糖尿病（OMIM:125853）	否
先天性糖基化缺陷	ALG1	先天性糖基化病 Ik 型（OMIM:608540）	否
先天性糖基化缺陷	ALG11	先天性糖基化病 Ip 型（OMIM:613661）	否

先天性糖基化缺陷	ALG12	先天性糖基化病 Ig 型（OMIM:607143）	否
先天性糖基化缺陷	ALG13	先天性糖基化病 Is 型（OMIM:300884）	否
先天性糖基化缺陷	ALG2	先天性糖基化病 Ii 型（OMIM:607906）	否
先天性糖基化缺陷	ALG3	先天性糖基化病 Id 型（OMIM:601110）	否
先天性糖基化缺陷	ALG6	先天性糖基化病 Ic 型（OMIM:603147）	否
先天性糖基化缺陷	ALG8	先天性糖基化病 Ih 型（OMIM:608104）	否
先天性糖基化缺陷	ALG9	先天性糖基化病 Il 型（OMIM:608776）	否
先天性糖基化缺陷	B4GALT1	先天性糖基化病 IId 型（OMIM:607091）	否
先天性糖基化缺陷	CCDC115	先天性糖基化病 IIo 型（OMIM:616828）	否
先天性糖基化缺陷	COG1	先天性糖基化病 IIg 型（OMIM:611209）	否
先天性糖基化缺陷	COG2	先天性糖基化的障碍 Iiq 型（OMIM:617395）	否
先天性糖基化缺陷	COG4	先天性糖基化病 IIj 型（OMIM:613489）	否
先天性糖基化缺陷	COG5	先天性糖基化病 IIi 型（OMIM:613612）	否
先天性糖基化缺陷	COG6	先天性糖基化病 IIl 型（OMIM:614576）	否
先天性糖基化缺陷	COG7	先天性糖基化病 IIe 型（OMIM:608779）	否
先天性糖基化缺陷	COG8	先天性糖基化病 IIh 型（OMIM:611182）	否
先天性糖基化缺陷	DDOST	先天性糖基化病 Ir 型（OMIM:614507）	否
先天性糖基化缺陷	DOLK	先天性糖基化病 Im 型（OMIM:610768）	否
先天性糖基化缺陷	DPAGT1	先天性糖基化病 Ij 型（OMIM:608093）	否
先天性糖基化缺陷	DPM1	先天性糖基化病 Ie 型（OMIM:608799）	否
先天性糖基化缺陷	DPM2	先天性糖基化病 Iu 型（OMIM:615042）	否
先天性糖基化缺陷	DPM3	先天性糖基化病 Io 型（OMIM:612937）	否
先天性糖基化缺陷	MGAT2	先天性糖基化病 IIa 型（OMIM:212066）	否
先天性糖基化缺陷	MOGS	先天性糖基化病 IIb 型（OMIM:606056）	否
先天性糖基化缺陷	MPDU1	先天性糖基化病 If 型（OMIM:609180）	否
先天性糖基化缺陷	MPI	先天性糖基化病 Ib 型（OMIM:602579）	否
先天性糖基化缺陷	NGLY1	先天性糖基化病 Iv 型（OMIM:615273）	否
先天性糖基化缺陷	PMM2	先天性糖基化病 Ia 型（OMIM:212065）	否
先天性糖基化缺陷	RFT1	先天性糖基化病 In 型（OMIM:612015）	否
先天性糖基化缺陷	SLC35A1	先天性糖基化病 IIf 型（OMIM:603585）	否
先天性糖基化缺陷	SLC35A2	先天性糖基化病 IIm 型（OMIM:300896）	否
先天性糖基化缺陷	SLC35C1	先天性糖基化病 IIc 型（OMIM:266265）	否
先天性糖基化缺陷	SLC39A8	先天性糖基化病 IIn 型（OMIM:616721）	否
先天性糖基化缺陷	SRD5A3	先天性糖基化病 Iq 型（OMIM:612379）	否
先天性糖基化缺陷	STT3A	先天性糖基化病 Iw 型（OMIM:615596）	否
先天性糖基化缺陷	STT3B	先天性糖基化病 Ix 型（OMIM:615597）	否
先天性糖基化缺陷	TMEM165	先天性糖基化病 IIk 型（OMIM:614727）	否
先天性糖基化缺陷	TMEM199	先天性糖基化病 IIp 型（OMIM:616829）	否
线粒体（核基因）相关	ATPAF2	线粒体复合体 V 缺乏症，核型 1（OMIM:604273）	否
线粒体（核基因）相关	BCS1L	线粒体复合体 III 缺乏症，核型 1（OMIM:124000）	否
线粒体（核基因）相关	BOLA3	多线粒体功能障碍综合征 2 型（OMIM:614299）	否
线粒体（核基因）相关	COA7	线粒体复合体 IV 缺乏症（OMIM:220110）	否

线粒体（核基因）相关	COA8	线粒体复合体 IV 缺乏症（OMIM:220110）	否
线粒体（核基因）相关	COX10	线粒体复合体 IV 缺乏症（OMIM:220110）	否
线粒体（核基因）相关	COX14	线粒体复合体 IV 缺乏症（OMIM:220110）	否
线粒体（核基因）相关	COX15	Leigh 综合征（OMIM:256000）	否
线粒体（核基因）相关	COX20	线粒体复合体 IV 缺乏症（OMIM:220110）	否
线粒体（核基因）相关	COX6B1	线粒体复合体 IV 缺乏症（OMIM:220110）	否
线粒体（核基因）相关	COX8A	线粒体复合体 IV 缺乏症（OMIM:220110）	否
线粒体（核基因）相关	CYC1	线粒体复合体 III 缺乏症，核型 6（OMIM:615453）	否
线粒体（核基因）相关	DGUOK	线粒体 DNA 缺失综合征 3 型（OMIM:251880）	否
线粒体（核基因）相关	FASTKD2	线粒体复合体 IV 缺乏症（OMIM:220110）	否
线粒体（核基因）相关	FBXL4	线粒体 DNA 缺失综合征 13 型（OMIM:615471）	否
线粒体（核基因）相关	FOXRED1	Leigh 综合征（OMIM:256000）	否
线粒体（核基因）相关	KYNU	羟基犬尿酸尿症（OMIM:236800）	否
线粒体（核基因）相关	LRPPRC	法国-加拿大型 Leigh 综合征（OMIM:220111）	否
线粒体（核基因）相关	LYRM7	线粒体复合体 III 缺乏症，核型 8（OMIM:615838）	否
线粒体（核基因）相关	MGME1	线粒体 DNA 缺失症 11 型	否
线粒体（核基因）相关	MPV17	线粒体 DNA 缺失综合征 6 型（OMIM:256810）	否
线粒体（核基因）相关	NDUFA10	Leigh 综合征（OMIM:256000）	否
线粒体（核基因）相关	NDUFA12	Leigh 综合征（OMIM:256000）	否
线粒体（核基因）相关	NDUFA4	Leigh 综合征（PMID 23746447）	否
线粒体（核基因）相关	NDUFA9	Leigh 综合征（OMIM:256000）	否
线粒体（核基因）相关	NDUFAF2	Leigh 综合征（OMIM:256000）	否
线粒体（核基因）相关	NDUFAF6	Leigh 综合征（OMIM:256000）	否
线粒体（核基因）相关	NDUFS3	Leigh 综合征（OMIM:256000）	否
线粒体（核基因）相关	NDUFS7	Leigh 综合征（OMIM:256000）	否
线粒体（核基因）相关	NDUFS8	Leigh 综合征（OMIM:256000）	否
线粒体（核基因）相关	PDHA1	X 连锁 Leigh 综合征	否
线粒体（核基因）相关	PET100	线粒体复合体 IV 缺乏症（OMIM:220110）	否
线粒体（核基因）相关	POLG	线粒体 DNA 缺失综合征 4A 型（OMIM:203700）	否
线粒体（核基因）相关	POLG2	常染色体显性进行性外眼肌瘫痪伴线粒体 DNA 缺失 4 型（OMIM:610131）	否
线粒体（核基因）相关	RRM2B	线粒体 DNA 缺失综合征 8A 型（OMIM:612075）	否
线粒体（核基因）相关	SCO1	线粒体复合体 IV 缺乏症（OMIM:220110）	否
线粒体（核基因）相关	SDHA	Leigh 综合征（OMIM:256000）	否
线粒体（核基因）相关	SDHAF1	线粒体复合体 II 缺乏症（OMIM:252011）	否
线粒体（核基因）相关	SDHD	线粒体复合体 II 缺乏症（OMIM:252011）	否
线粒体（核基因）相关	SLC25A4	线粒体 DNA 缺失综合征 12 型（OMIM:615418）	否
线粒体（核基因）相关	SUCLA2	线粒体 DNA 缺失综合征 5 型（OMIM:612073）	否
线粒体（核基因）相关	SUCLG1	线粒体 DNA 缺失综合征 9 型（OMIM:245400）	否
线粒体（核基因）相关	SURF1	Leigh 综合征（OMIM:256000）	否
线粒体（核基因）相关	TACO1	线粒体复合体 IV 缺乏症（OMIM:220110）	否
线粒体（核基因）相关	TFAM	线粒体 DNA 缺失综合征 15（肝脑型）（OMIM:617156）	否

线粒体（核基因）相关	TK2	线粒体 DNA 缺失综合征 2 型（OMIM:609560）	否
线粒体（核基因）相关	UQCC2	线粒体复合体 III 缺乏症，核型 7（OMIM:615824）	否
线粒体（核基因）相关	UQCRB	线粒体复合体 III 缺乏症，核型 3（OMIM:615158）	否
线粒体（核基因）相关	UQCRC2	线粒体复合体 III 缺乏症，核型 5（OMIM:615160）	否
线粒体（核基因）相关	UQCRQ	线粒体复合体 III 缺乏症，核型 4（OMIM:615159）	否
易感	CCR5	丙肝病毒易感型（OMIM:609532）	是
易感	IFNAR2	{乙肝病毒易感型}（OMIM:610424）	是
易感	IFNGR1	{乙肝病毒易感型}（OMIM:610424）	是
易感	IFNL3	丙肝病毒易感型（OMIM:609532）	是
易感	IL10RB	{乙肝病毒易感型}（OMIM:610424）	是
易感	IL18BP	肝炎及爆发性病毒易感性（OMIM:618549）	是
有机酸代谢异常	ETFA	戊二酸血症 2 型（OMIM:231680）	否
有机酸代谢异常	ETFB	戊二酸血症 2 型（OMIM:231680）	否
有机酸代谢异常	ETFDH	戊二酸血症 2 型（OMIM:231680）	否
有机酸代谢异常	FH	延胡索酸酶缺乏症（OMIM:606812）	否
有机酸代谢异常	MCEE	甲基丙二酸单酰-coa 表异构酶缺乏症（OMIM:251120）	否
有机酸代谢异常	MMAA	CblA 型甲基丙二酸血症（OMIM:251100）	否
有机酸代谢异常	MMAB	CblA 型甲基丙二酸血症（OMIM:251100）	否
有机酸代谢异常	MMACHC	甲基丙二酸尿症伴同型半胱氨酸尿症 CblC 型（OMIM:277400）	否
有机酸代谢异常	MMADHC	CblD 型甲基丙二酸尿症伴同型胱氨酸尿症（OMIM:277410）	否
有机酸代谢异常	MUT	甲基丙二酸尿症 mut(0)型（OMIM:251000）	否
有机酸代谢异常	MVK	甲羟戊酸尿症（OMIM:610377）	否
有机酸代谢异常	PC	丙酮酸羧化酶缺乏症（OMIM:266150）	否
有机酸代谢异常	PCCA	丙酸血症（OMIM:606054）	否
有机酸代谢异常	PCCB	丙酸血症（OMIM:606054）	否
有机酸代谢异常	SERAC1	3-甲基戊烯二酸尿症伴耳聋、脑病及 leigh 样综合征（OMIM:614739	否
脂类代谢缺陷	ABCA1	高密度脂蛋白缺乏症（OMIM:205400）	否
脂类代谢缺陷	ABCG5	谷固醇血症（OMIM:210250）	否
脂类代谢缺陷	ABCG8	谷固醇血症（OMIM:210250）	否
脂类代谢缺陷	ABHD5	Chanarin-Dorfman 综合征（OMIM:275630）	否
脂类代谢缺陷	ACAD9	乙酰 CoA 脱氢酶缺乏症（OMIM:611126）	否
脂类代谢缺陷	ACADM	中链酰基辅酶 A 脱氢酶缺乏症（OMIM:201450）	否
脂类代谢缺陷	ACADVL	极长链酰基辅酶 A 脱氢酶缺乏症（OMIM:201475）	否
脂类代谢缺陷	AGPAT2	先天性全身脂肪营养不良 1 型（OMIM:608594）	否
脂类代谢缺陷	ANGPTL3	家族性低β脂蛋白血症 2 型（OMIM:605019）	否
脂类代谢缺陷	APOA1	家族性高密度脂蛋白缺乏症（OMIM:604091）	否
脂类代谢缺陷	APOA5	高脂蛋白血症 V 型（OMIM:144650）	否
脂类代谢缺陷	APOE	脂蛋白肾小球病（OMIM:611771）	否
脂类代谢缺陷	BSCL2	先天性全身脂肪营养不良 2 型（OMIM:269700）	否
脂类代谢缺陷	CAVIN1	先天性全身脂肪营养不良 4 型（OMIM:613327）	否
脂类代谢缺陷	CPT1A	肉碱软脂酰转移酶 1 缺乏症（OMIM:255120）	否
脂类代谢缺陷	CPT2	严重婴儿型肉碱软脂酰转移酶 2 缺乏症（OMIM:600649）	否

脂类代谢缺陷	GPD1	暂时性婴儿高甘油三酯血症（OMIM:614480）	否
脂类代谢缺陷	HADHA	三官能团蛋白缺乏症（OMIM:609015）	否
脂类代谢缺陷	HADHB	三官能团蛋白缺乏症（OMIM:609015）	否
脂类代谢缺陷	LIPA	胆固醇酯沉积病（OMIM:278000）	否
脂类代谢缺陷	LIPI	家族性高甘油三酯血症（OMIM:145750）	否
脂类代谢缺陷	LIPT1	脂蛋白转移酶缺乏症（OMIM:616299）	否
脂类代谢缺陷	LPL	家族性复合高脂血症（OMIM:144250）	否
脂类代谢缺陷	MTTP	无β脂蛋白血症（OMIM:200100）	否
脂类代谢缺陷	PNPLA2	中性脂质贮积病伴肌病（OMIM:610717）	否
脂类代谢缺陷	SAR1B	乳糜微粒滞留病（OMIM:246700）	否
脂类代谢缺陷	SLC22A5	原发性肉碱缺乏症（OMIM:212140）	否
脂类代谢缺陷	SLC25A20	肉碱酰基转移酶缺乏症（OMIM:212138）	否
脂质代谢缺陷	LIPC	肝脂酶缺乏症（OMIM:614025）	是
其他	ABCD1	X 连锁肾上腺脑白质营养不良症（OMIM:300100）	否
其他	ACVRL1	遗传性出血性毛细血管扩张症 2 型（OMIM:600376）	否
其他	ADA2	Sneddon 综合征（OMIM:182410）	否
其他	ADAMTSL2	Geleophysic 发育不良 1 型（OMIM:231050）	否
其他	AGPS	肢根点状软骨发育不良 3 型（OMIM:600121）	否
其他	AGXT	原发性高草酸尿症 1 型（OMIM:259900）	否
其他	AIRE	自身免疫性多发内分泌腺病综合征 1 型（OMIM:240300）	否
其他	ALDH2	急性酒精敏感症（OMIM:610251）	否
其他	ALMS1	Alstrom 综合征（OMIM:203800）	否
其他	ANK1	球形红细胞增多症 1 型（OMIM:182900）	否
其他	ANKS6	肾单位肾痨 16 型（OMIM:615382）	否
其他	AP1S1	MEDNIK 综合征（OMIM:609313）	否
其他	ASAH1	脊肌萎缩症伴进行性肌阵挛性癫痫（OMIM:159950）	否
其他	ATP13A2	Kufor-Rakeb 综合征（OMIM:606693）	否
其他	ATP6AP1	免疫缺陷症 47 型（OMIM:300972）	否
其他	B2M	免疫缺陷症 43 型（OMIM:241600）	否
其他	B9D2	Meckel 综合征 10 型（OMIM:614175）	是
其他	BCAP31	耳聋、肌张力障碍和大脑脱髓鞘（OMIM:300475）	否
其他	BMP2	短指（趾）畸形 A2 型（OMIM:112600）	否
其他	BMPER	透明脊椎异骨症（OMIM:608022）	否
其他	BRAF	努南综合征 7 型（OMIM:613706）	否
其他	CALM1	长 QT 综合征 14 型（OMIM:616247）	否
其他	CALM2	长 QT 综合征 15 型（OMIM:616249）	否
其他	CALM3	磷酸化酶激酶缺乏症	否
其他	CASP10	自身免疫性淋巴细胞增生综合征 2A 型（OMIM:603909）	否
其他	CCND1	结直肠癌（OMIM:114500）	否
其他	CD40LG	X 连锁高 IgM 综合征（OMIM:308230）	否
其他	CDAN1	先天性红细胞生成障碍性贫血 1a 型（OMIM:224120）	否
其他	CDIN1	先天性红细胞生成异常性贫血 1b 型（OMIM:615631）	是

其他	CDKN1C	Beckwith-Wiedemann 综合征（OMIM:130650）	否
其他	CEP164	肾单位肾痨 15 型（OMIM:614845）	否
其他	CEP19	病态肥胖症和精子生成障碍（OMIM:615703）	否
其他	CEP83	肾单位肾痨 18 型（OMIM:615862）	否
其他	CFAP53	运动纤毛的组装和功能中的关键作用，突变导致先天性心脏病。（PMID: 26538025）	否
其他	CFC1	内脏异位 2 型（OMIM:605376）	否
其他	CIITA	裸淋巴细胞综合征 2 型（OMIM:209920）	否
其他	CLPB	3-甲基戊烯二酸尿症伴白内障、神经病及中性粒细胞减少（OMIM:616271）	是
其他	CTC1	脑视网膜微血管病伴钙化囊肿（OMIM:612199）	否
其他	CTLA4	自身免疫性淋巴细胞增生综合征 5 型（OMIM:616100）	否
其他	CTNS	肾病型胱氨酸症（OMIM:219800）	否
其他	CTSC	Papillon-Lefevre 综合征（OMIM:245000）	否
其他	CYBA	常染色体隐性细胞色素 B 阴性慢性肉芽肿病（OMIM:233690）	否
其他	CYP27A1	脑腱黄瘤病（OMIM:213700）	否
其他	CYP3A4	维生素 D 依赖性佝偻病 3 型（omim:619073）	是
其他	DCDC2	肾单位肾痨 19 型（OMIM:616217）	否
其他	DCLRE1C	Omenn 综合征（OMIM:603554）	否
其他	DHCR7	Smith-Lemli-Opitz 综合征（OMIM:270400）	否
其他	DHDDS	视网膜色素变性 59 型（OMIM:613861）	否
其他	DKC1	X 连锁先天性角化不良 6 型（OMIM:305000）	否
其他	DLG4	智力发育障碍 62 型;MRD62（OMIM:618793）	是
其他	DYNC2LI1	多指性发育不全（OMIM:617088）	否
其他	ENG	遗传性出血性毛细血管扩张症 1 型（OMIM:187300）	否
其他	EPM2A	Lafora 病（OMIM:254780）	否
其他	EXTL3	免疫骨骼发育不良伴神经发育异常（OMIM:617425）	否
其他	F8	甲型血友病（OMIM:306700）	否
其他	F9	乙型血友病（OMIM:306900）	否
其他	FADD	复发感染、脑病、肝功能不全及心血管畸形（OMIM:613759）	否
其他	FAM111A	细薄骨发育不良（OMIM:602361）	否
其他	FAN1	巨核间质性肾炎（OMIM:614817）	否
其他	FAS	自身免疫性淋巴细胞增生综合征 1 型（OMIM:601859）	否
其他	FASLG	自身免疫性淋巴细胞增生综合征 1 型（OMIM:601859）	否
其他	FASN	癫痫性脑病	是
其他	FCGR2A	疟疾易感型（OMIM:611162）	否
其他	FERMT3	白细胞粘附缺陷 3 型（OMIM:612840）	否
其他	FGB	先天性纤维蛋白原缺乏血症（OMIM:202400）	否
其他	FGG	先天性纤维蛋白原缺乏血症（OMIM:202400）	否
其他	FIG4	家族性肌萎缩性侧索硬化症 11 型（OMIM:612577）	否
其他	FXN	Friedreich 共济失调（OMIM:229300）	否
其他	G6PC3	严重先天性中性白细胞减少症 4 型（OMIM:612541）	否

其他	GALC	Krabbe 病（OMIM:245200）	否
其他	GANAB	多囊肾病 3 型（OMIM:600666）	否
其他	GATA6	法洛四联症（OMIM:187500）	否
其他	GDF1	法洛四联症（OMIM:187500）	否
其他	GDF2	遗传性出血性毛细血管扩张症 5 型（OMIM:615506）	否
其他	GLIS2	肾单位肾痨 7 型（OMIM:611498）	否
其他	GLUD1	家族性高胰岛素血症 6 型（OMIM:606762）	否
其他	GNPAT	肢根点状软骨发育不良 2 型（OMIM:222765）	否
其他	GUCY2D	Leber 氏先天性黒蒙 1 型（OMIM:204000）	否
其他	H19	Beckwith-Wiedemann 综合征（OMIM:130650）	否
其他	HBB	β地中海贫血（OMIM:613985）	否
其他	HEXA	Tay-Sachs 病（OMIM:272800）	否
其他	HLA-DRB1	易感性多发性硬化 1 型（OMIM:126200）	否
其他	HNRNPH2	X 连锁贝恩型智力发育迟缓综合征（OMIM:300986）	是
其他	IARS1	婴儿肝病（OMIM：617093）	否
其他	IDUA	Hurler-Scheie 综合征（OMIM:607015）	否
其他	IFT81	视网膜营养不良和脑部病变（PMID: 26275418）	否
其他	IL21R	Il21R 相关免疫缺陷症（OMIM:615207）	否
其他	IL7R	B 细胞和 NK 细胞阳性、T 细胞阴性的重症联合免疫缺陷（OMIM:608971）	否
其他	INVS	肾单位肾痨 2 型（OMIM:602088）	否
其他	ITCH	多系统自身免疫病伴面部畸形（OMIM:613385）	否
其他	ITK	淋巴增殖综合征 1 型（OMIM:613011）	否
其他	KCNH1	Temple-Baraitser 综合征（OMIM:611816）	否
其他	KCNQ1	Jervell 和 Lange-Nielsen 综合征 1 型（OMIM:220400）	是
其他	KCTD7	进行性肌阵挛性癫痫 3 型（OMIM:611726）	否
其他	KRAS	努南综合征 3 型（OMIM:609942）	否
其他	LARS2	Perrault 综合征 4 型（OMIM:615300）	否
其他	LBR	Reynolds 综合征（OMIM:613471）	否
其他	LMNA	Slovenian 型心-手综合征（OMIM:610140）	否
其他	LZTR1	努南综合征 10 型（OMIM:616564）	否
其他	MDH2	早发幼儿癫痫性脑病 51 型（OMIM:617339）	否
其他	MEGF8	Carpenter 综合征 2 型（OMIM:614976）	否
其他	MMP21	内脏异位 7 型（OMIM:616749）	否
其他	MTHFD1	叶酸敏感性脊柱裂，易感性（OMIM:601634）	否
其他	MVD	多形性汗孔角化症 7 型（OMIM:614714）	是
其他	NAGA	α-N-乙酰氨基半乳糖转移酶缺乏症（OMIM:609241）	否
其他	NAT2	慢乙酰化作用（OMIM:243400）	否
其他	NBAS	小儿肝功能衰竭综合征 2 型（OMIM:616483）	否
其他	NCF1	常染色体隐性细胞色素 B 阳性慢性肉芽肿病 1 型（OMIM:233700）	否
其他	NCF2	常染色体隐性细胞色素 B 阳性慢性肉芽肿病 2 型（OMIM:233710）	否
其他	NEK8	肾单位肾痨 9 型（OMIM:613824）	否

其他	NEK9	致死性先天性挛缩综合征 10 型（OMIM:617022）	是
其他	NHLRC1	Lafora 病（OMIM:254780）	否
其他	NHP2	常染色体隐性先天性角化不良 2 型（OMIM:613987）	否
其他	NOP10	常染色体隐性先天性角化不良 1 型（OMIM:224230）	否
其他	NPHP1	肾单位肾痨 1 型（OMIM:256100）	否
其他	NPHP3	肾单位肾痨 3 型（OMIM:604387）	否
其他	NPHP4	肾单位肾痨 4 型（OMIM:606966）	否
其他	NSMCE2	Seckel syndrome 10 (OMIM:617253)	否
其他	OCLN	带状钙化伴简化性迂回及多小脑回畸形（OMIM:251290）	否
其他	OFD1	Joubert 综合征 10 型（OMIM:300804）	否
其他	PGK1	磷酸甘油酸激酶缺乏症（OMIM:300653）	否
其他	PHYH	雷夫叙姆病（OMIM:266500）	否
其他	PIEZO1	遗传性淋巴水肿 III 型（OMIM:616843）	否
其他	PIGA	先天性多发畸形-张力减退-癫痫发作综合征 2 型（OMIM:300868）	否
其他	PKD1	常染色体显性多囊肾病 1 型（OMIM:173900）	否
其他	PKD2	常染色体显性多囊肾病 2 型（OMIM:613095）	否
其他	PKHD1	常染色体隐性多囊肾病（OMIM:263200）	否
其他	POLD1	下颌骨发育不全、耳聋、类早老和脂肪营养不良综合征（OMIM:615381）	否
其他	POMC	肥胖（OMIM:601665）	否
其他	PRF1	家族性嗜血细胞性淋巴组织细胞增多症 2 型（OMIM:603553）	否
其他	PRKD1	先天性心脏缺陷和外胚层发育不良（OMIM:617364）	是
其他	PSMB8	自身炎症反应、脂质营养不良及皮肤病综合征（OMIM:256040）	否
其他	PTF1A	胰腺发育不全 2 型（OMIM:615935）	否
其他	PTPN11	努南综合征 1 型（OMIM:163950）	否
其他	PTPRC	B 细胞和 NK 细胞阳性、T 细胞阴性的重症联合免疫缺陷（OMIM:608971）	否
其他	RAF1	努南综合征 5 型（OMIM:611553）	否
其他	RAG1	RAG1 相关重型联合免疫缺陷症（OMIM:601457）	否
其他	RAG2	RAG1 相关重型联合免疫缺陷症（OMIM:601457）	否
其他	RBCK1	葡聚糖体肌病 1 型伴或无免疫缺陷（OMIM:615895）	否
其他	RFX5	裸淋巴细胞综合征 2 型（OMIM:209920）	否
其他	RFXANK	裸淋巴细胞综合征 2 型（OMIM:209920）	否
其他	RFXAP	裸淋巴细胞综合征 2 型（OMIM:209920）	否
其他	RHAG	调节型 Rh-null 症（OMIM:268150）	否
其他	RINT1	婴儿肝功能衰竭综合征 3;ILFS3（OMIM:618641）	是
其他	RIT1	努南综合征 8 型（OMIM:615355）	否
其他	RNASEH2A	Aicardi-Goutieres 综合征 4 型（OMIM:610333）	否
其他	SAMHD1	Aicardi-Goutieres 综合征 5 型（OMIM:612952）	否
其他	SC5D	烷醇症（OMIM:607330）	否
其他	SCN9A	早发幼儿癫痫性脑病 6 型（OMIM:607208）	否
其他	SCP2	脑白质病伴张力失调及运动神经病（OMIM:613724）	否

其他	SCYL1	常染色体隐性脊髓小脑性共济失调 21 型（OMIM:616719）	否
其他	SERPIND1	肝素辅助因子 II 缺乏症（OMIM:612356）	是
其他	SH2D1A	X 连锁淋巴增殖综合征 1 型（OMIM:308240）	否
其他	SHANK3	Phelan-McDermid 综合征（OMIM:606232）	否
其他	SKIV2L	毛发、肝及小肠综合征 2 型（OMIM:614602）	否
其他	SLC10A1	NTCP 缺乏症（PMID：24867799）	否
其他	SLC29A3	组织细胞增多-淋巴结病综合征（OMIM:602782）	否
其他	SLC52A1	核黄素缺乏症（omim:615026）	是
其他	SLC52A3	Brown-Vialetto-Van+Laere 综合征 1 型（OMIM:211530）	否
其他	SLC6A19	Hartnup 病（OMIM:234500）	否
其他	SOS1	努南综合征 4 型（OMIM:610733）	否
其他	SOS2	努南综合征 9 型（OMIM:616559）	否
其他	SPRTN	Ruijs-Aalfs 综合征（OMIM:616200）	否
其他	STN1	脑血管病变与钙化和囊肿 2 型（OMIM:617341）	否
其他	STX11	家族性嗜血细胞性淋巴组织细胞增多症 4 型（OMIM:603552）	否
其他	STXBP2	家族性嗜血细胞性淋巴组织细胞增多症 5 型（OMIM:613101）	否
其他	SYNJ1	帕金森病 20 型（OMIM:615530）	否
其他	TAZ	Barth 综合征（OMIM:302060）	否
其他	TCIRG1	常染色体隐性骨硬化症 1 型（OMIM:259700）	否
其他	TGFB1	进行性骨干发育不良（OMIM:131300）	否
其他	TRAF3IP1	Senior-Loken 综合征 9 型（OMIM:616629）	否
其他	TRAPPC11	肢带型肌营养不良 2S 型（OMIM:615356）	否
其他	TREX1	Aicardi-Goutieres 综合征 1 型（OMIM:225750）	否
其他	TRIM37	肌肝脑眼侏儒症（OMIM:253250）	否
其他	TTC21B	肾单位肾痨 12 型（OMIM:613820）	否
其他	TTC37	毛发、肝及小肠综合征 1 型（OMIM:222470）	否
其他	TTPA	共济失调伴维生素 E 缺乏症（OMIM:277460）	否
其他	TWNK	婴儿型脊髓小脑性共济失调（OMIM:271245）	否
其他	UBR1	Johanson-Blizzard 综合征（OMIM:243800）	否
其他	UNC13D	家族性嗜血细胞性淋巴组织细胞增多症 3 型（OMIM:608898）	否
其他	USP18	Pseudo-TORCH 综合征 2 型（OMIM:617397）	否
其他	VARS1	神经发育障碍伴畸形，癫痫发作和皮质萎缩（OMIM:617802）	是
其他	VHL	Von+Hipple-Lindau 病（OMIM:193300）	否
其他	WDR36	原发性开角型青光眼 1G 型（OMIM:609887）	是
其他	XPNPEP3	肾单位肾痨样肾病 1 型（OMIM:613159）	否
其他	YARS2	肌病、乳酸酸中毒及铁粒幼红细胞性贫血 2 型（OMIM:613561）	否
其他	ZFYVE19	-	是
其他	ZNF423	肾单位肾痨 14 型（OMIM:614844）	否
